# Effects of pyric herbivory on prairie-chicken (*Tympanuchus* spp) habitat

**DOI:** 10.1371/journal.pone.0234983

**Published:** 2020-06-23

**Authors:** Heath D. Starns, Samuel D. Fuhlendorf, Robert D. Elmore, Dirac Twidwell, Eric T. Thacker, Torre J. Hovick, Barney Luttbeg

**Affiliations:** 1 Department of Natural Resource Ecology and Management, Oklahoma State University, Stillwater, Oklahoma, United States of America; 2 Department of Agronomy and Horticulture, University of Nebraska-Lincoln, Lincoln, Nebraska, United States of America; 3 S.J. and Jessie E. Quinney College of Natural Resources, Utah State University, Logan, Utah, United States of America; 4 School of Natural Resource Sciences, North Dakota State University, Fargo, North Dakota, United States of America; 5 Department of Integrative Biology, Oklahoma State University, Stillwater, Oklahoma, United States of America; University of Illinois at Urbana-Champaign, UNITED STATES

## Abstract

The reduction and simplification of grasslands has led to the decline of numerous species of grassland fauna, particularly grassland-obligate birds. Prairie-chickens (*Tympanuchus* spp.) are an example of obligate grassland birds that have declined throughout most of their distribution and are species of conservation concern. Pyric herbivory has been suggested as a land management strategy for enhancing prairie-chicken habitat and stabilizing declining population trends. We assessed differences in vegetation structure created by pyric herbivory compared to fire-only treatments to determine whether pyric herbivory increased habitat heterogeneity for prairie-chickens, spatially or temporally. Our study was performed at four sites in the southern Great Plains, all within the current or historic distribution of either lesser (*T*. *pallidicinctus*), greater (*T*. *cupido*), or Attwater’s (*T*. *cupido attwateri*) prairie-chickens. Key vegetation characteristics of grass cover and vegetation height in pyric herbivory and fire-only treatments were within the recommended range of values for prairie-chickens during their distinct life history stages. However, patches managed via pyric herbivory provided approximately 5% more forb cover than fire-only treatments for almost 30 months post-fire. Additionally, pyric herbivory extended the length of time bare ground was present after fires. Pyric herbivory also reduced vegetation height and biomass, with mean vegetation height in pyric herbivory treatments lagging behind fire-only treatments by approximately 15 months. Canopy cover in fire-only treatments exceeded levels recommended for prairie-chicken young within 12 months post-fire. However, canopy cover in pyric herbivory treatments never exceeded the maximum recommended levels. Overall, it appears that pyric herbivory improves vegetation characteristics reported as critical to prairie-chicken reproduction. Based on our results, we suggest pyric herbivory as a viable management technique to promote prairie-chicken habitat in the southern Great Plains, while still accommodating livestock production.

## Introduction

Over the past century, rangelands (grasslands, shrublands, and savannas) have declined worldwide, primarily as a result of anthropogenic land use change [1]. Rangelands that persist often suffer from fragmentation, which is driven by many factors, including urbanization, conversion to croplands, and energy development [2]. Furthermore, in the Great Plains of North America, past rangeland management practices have also reduced the quality of remaining habitat for prairie-chickens [3, 4].

Departure from historical fire regimes, as well as decoupling of the historical interaction between fire and grazing contributed to this degradation [4, 5]. In areas where fire frequency increased (large-scale annual burning in the Flint Hills of Kansas), structural and compositional vegetation characteristics became simplified, often with a net result of lower biodiversity [4, 6, 7]. Areas where fire frequency decreased (virtually the entirety of the Great Plains with the exception of the Flint Hills) were invaded by woody species [4–8]. As a consequence, woody encroachment led to population declines in numerous grassland fauna, particularly grassland birds [9–11]. While some grassland bird species require vegetation that has been recently disturbed, others select for long-undisturbed vegetation [9]. Therefore, evidence suggests a wide range of vegetation structure is essential for conservation of this suite of birds [12–14].

Prairie-chickens (*Tympanuchus* spp.) have been suggested as indicator species for rangeland conservation [13–15] because they require heterogeneous vegetation structure to carry out the distinct stages of their life history [16–19]. Furthermore, of the five *Tympanuchus* species and subspecies, only one (*T*. *phasianellus*) is currently considered a species of “least concern” according to the International Union for Conservation of Nature and Natural Resources [20]. Of the others, one (*T*. *c*. *cupido*) is extinct, one (*T*. *c*. *attwateri*) is critically endangered, and two (*T*. *pallidicinctus* and *T*. *c*. *pinnatus*) are considered vulnerable. While prairie-chickens historically occupied most of the southern Great Plains, fragmentation and habitat loss have significantly reduced their occupied ranges [21].

In general, prairie-chickens require varied vegetation structure throughout their lifespan. They typically select for bare ground or short vegetation to carry out their mating display on areas of communal courtship known as leks [19, 22, 23]. For nesting cover, lesser prairie-chickens in mixed-grass/shrub communities select nest sites in low-growing shrubs [24] such as sand shinnery oak (*Quercus havardii*) or sand sagebrush (*Artemisia filifolia*). In mixed-grass prairies without shrubs, lesser prairie-chickens often nest in areas of high grass residual cover from previous years of growth [25] similar to nesting cover of greater and Attwater’s prairie-chicken [26, 27]. Greater prairie-chickens select for nest sites with higher litter cover, which is associated with increased nest success [26]. Prairie-chickens also exhibit a general avoidance of trees throughout the year [16, 26, 28], with a decrease in probability of use by lesser prairie-chickens as density of trees per hectare increases [29]. Similarly, assessments of greater prairie-chicken habitat use revealed distance to nearest tree as one of the most influential factors in determining nest location [26]. Moreover, grasslands must vary in vegetation structure and functional group composition across time and space to provide habitat characteristics required by prairie-chickens for each distinct life stage [16, 17, 19].

Current recommendations for prairie-chicken conservation include practices that promote heterogeneity in grass or shrub cover and composition across the landscape and limit tree cover [16, 17]. Such heterogeneity can be spatial and/or temporal, and the combination of these often results in a “shifting mosaic,” which is a net benefit for biological diversity [9, 12, 30–32]. This shifting mosaic can occur at multiple spatial scales [16] and result from differential application of one or several management practices. For example, prescribed fire can be used to limit woody plants but also promote vegetation heterogeneity, commonly referred to as “pyrodiversity” [33]. Pyrodiversity provides variable vegetation structure and composition as a result of variation in fire regimes—fires occurring in different seasons and at different frequencies, intensities, and sizes across a landscape [33]. However, pyrodiversity often ignores effects of grazing. A heterogeneous vegetation mosaic can also be achieved by restoration of the complex spatial and temporal interaction between fire and herbivory [4, 7]. This interaction, termed pyric-herbivory [31], occurs as a result of free-roaming herbivores preferentially foraging on the most recently burned patches within a landscape [34]. Although the historical interaction occurred between bison (*Bison bison*) and fire, domestic grazers such as cattle (*Bos taurus*) exhibit similar responses to fire [34, 35]. Patches with greater time-since-fire are largely ignored by grazers, which allows for biomass accumulation that will fuel future fires [34, 36]. Landscape heterogeneity promoted by pyric-herbivory has been shown to increase bird diversity and change abundances of breeding birds in tallgrass prairie [9, 32].

The presence of three prairie grouse species in the southern Great Plains, combined with the availability of literature on their ecology, offer an opportunity to investigate generalized patterns affecting their conservation across multiple ecosystems within the region. Considering the wide range of vegetation structure required by prairie-chickens (Table 1) and their status as indicator species for grassland conservation, we sought to evaluate the potential for pyric-herbivory to create a shifting mosaic of vegetation comprised of lekking, nesting, and brooding cover across rangelands in the southern Great Plains. Our specific objectives were to (1) evaluate effects of time-since-fire and pyric-herbivory on plant functional group composition; (2) examine effects of time-since-fire and pyric-herbivory on vegetation structure; (3) determine whether pyric-herbivory results in plant communities that offer the structure and functional group composition necessary for prairie-chickens to complete their various life stages; and (4) determine whether pyric-herbivory affects invertebrate diversity or biomass compared to fire-only (pyrodiversity) treatments. To assess the shifting mosaic both spatially and temporally, we developed a large-scale experiment on sites within the historical distributions of lesser, greater, and Attwater’s prairie-chickens across the diverse ecoregions of the southern Great Plains.

**Table 1:** Prairie-chicken habitat requirements.

Species	Plant community	Habitat type	Grass cover (%)	Forb cover (%)	Shrub cover (%)	Bare ground (%)	Litter cover (%)	Grass height (cm)	Shrub Height (cm)	Source
ATPC	Gulf coastal prairie	Lekking	Nd	Nd	Nd	Nd	Nd	≤ 16	Nd	USFWS 2010
		Nesting	> 25	> 5	< 5	16.5	Nd	23–67.3	Nd	
		Brooding	> 25	> 5	< 5	Nd	Nd	10.1–50.8	Nd	
GRPC	Tallgrass prairie	Lekking	Nd	Nd	Nd	Nd	Nd	< 10	Nd	
		Nesting	> 25	> 5	Nd	Nd	Nd	26–50	Nd	
		Brooding	> 25	> 5	Nd	12	Nd	25–100	Nd	
LEPC	Sand shinnery oak	Lekking	Nd	Nd	Nd	41	17.1	< 10	Nd	Hagen et al 2013
		Nesting	8.9–16.7	.84–3.1	16.7–25.4	16.0–25.8	26.7–65.7	28.2–44.5	28.1–39.4	
		Brooding	10.6–19.8	1.7–5.5	14.6–25.8	28.8–43.4	32–44.5	18.8–29.3	23.1–34.6	
	Sand sagebrush	Lekking	Nd	Nd	Nd	Nd	Nd	Nd	Nd	
		Nesting	11.3–62.8	1.2–13.1	2.1–28.3	14.8–62	Nd	21–34.4	36.2–49.3	
		Brooding	7–19.3	8–20.7	5–11.4	Nd	Nd	Nd	Nd	
	Mixed-grass prairie	Lekking	Nd	Nd	Nd	Nd	Nd	Nd	Nd	Lautenbach 2017
		Nesting	65	20	Nd	20	< 10	15–25	Nd	
		Brooding	Nd	Nd	Nd	10	Nd	Nd	Nd	

Summary of habitat requirements for Attwater’s (ATPC), greater (GRPC), and lesser (LEPC) prairie-chickens and plant communities in which each are found. Nd = no data.

### Study sites and methods

**Fig 1:**
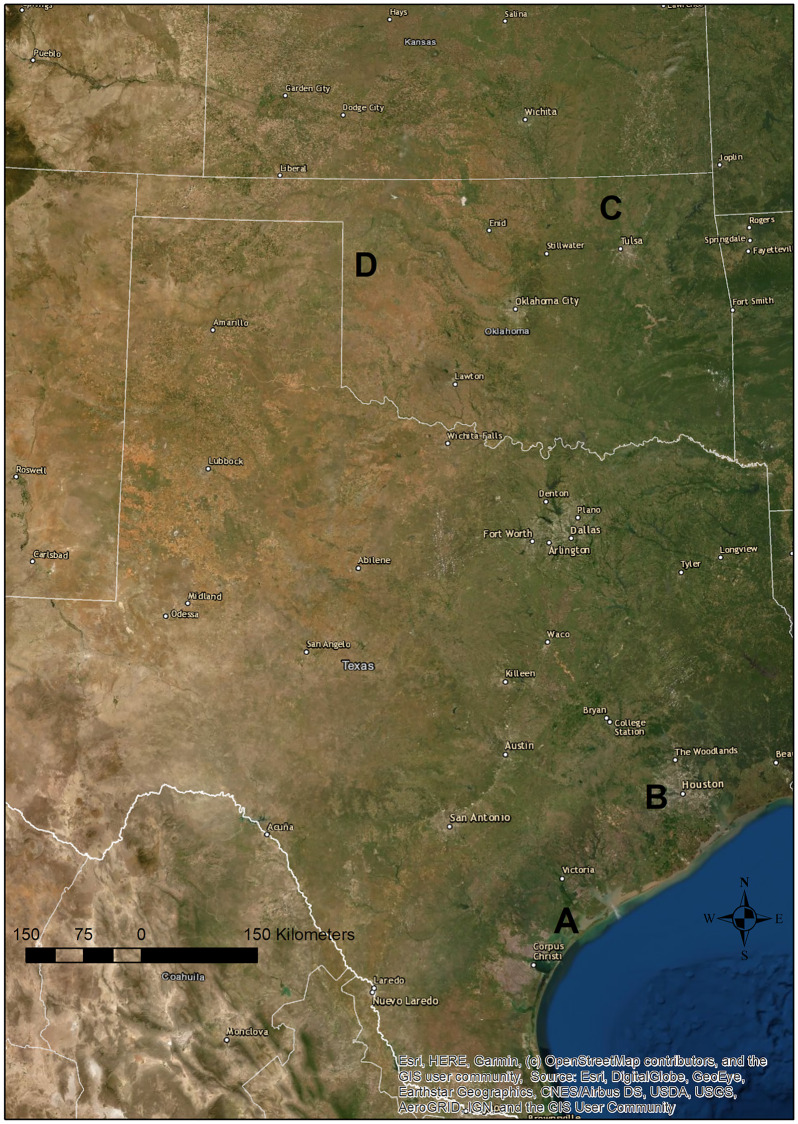
Study site map. Map showing approximate locations of each study site. A = Aransas National Wildlife Refuge (NWR); B = Attwater’s Prairie-chicken NWR; C = Tallgrass Prairie Preserve; D = Packsaddle Wildlife Management Area.

Data were collected from Aransas National Wildlife Refuge (Special Use Permit # 21530-15-06-DI), Attwater Prairie-chicken National Wildlife Refuge (Special Use Permit # ATW-14-004), Packsaddle Wildlife Management Area (verbal permission), and the Tallgrass Prairie Preserve (verbal permission). These four sites span much of the southern Great Plains (Fig 1) and are all within the current or historical distribution of the lesser, greater, or Attwater’s prairie-chicken: Aransas National Wildlife Refuge (NWR) in Texas (lat/long– 28° 18’ 29” N; 96° 48’ 17” W), Attwater’s Prairie-chicken NWR in Texas (lat/long– 29° 40’ 08” N; 96° 16’ 01” W), the Tallgrass Prairie Preserve in Oklahoma (lat/long– 36° 50’ 35” N; 96° 25’ 42” W), and Packsaddle Wildlife Management Area in Oklahoma (lat/long– 35° 53’ 38” N; 99° 39’ 41” W). All four sites were managed using fire to promote heterogeneity (e.g., pyrodiversity), and pyric-herbivory was a prominent part of the management strategy at three sites (Table 2). Due to specific management objectives and lack of infrastructure, Aransas NWR was entirely ungrazed by domestic herbivores. Burns were planned and carried out by personnel at each site according to site-specific management goals, but generally intended to mimic the timing and intensity of the pre-European fire regime. Because the goal was to promote heterogeneity, fires were allowed to be heterogeneous within and between patches, resulting in some patches burning incompletely as a result of fuel conditions and fire behavior, which further contribute to heterogeneity.

**Table 2:** Study sites and treatments.

Study Site	Size (ha)	State	Owner	Fire-only	Pyric-herbivory	MAP (cm)	Dominant Herbaceous Species	Dominant Woody Species	Patch size range (ha)
Aransas NWR	46,000	Texas	US Fish & Wildlife Service	Yes	No	105	*Schizachyrium scoparium*, *Sorghastrum nutans*, *Spartina spartinae*	*Prosopis glandulosa*, *Quercus virginiana*	60–500
Attwater’s Prairie-Chicken NWR	4,200	Texas	US Fish & Wildlife Service	Yes	Yes	111	*Schizachyrium scoparium*, *Sorghastrum nutans*, *Panicum virgatum*	NA	10–200
Packsaddle Wildlife Management Area	7,900	Oklahoma	Oklahoma Department of Wildlife Conservation	Yes	Yes	66	*Schizachyrium scoparium*, *Andropogon gerardii*, *Bouteloua curtipendula*	*Quercus havardii*, *Artemisia filifolia*	50–500
Tallgrass Prairie Preserve	16,000	Oklahoma	The Nature Conservancy	Yes	Yes	117	*Andropogon gerardii*, *Schizachyrium scoparium*, *Sorghastrum nutans*	*Quercus marilandica*, *Q*. *stellata*	40–750

Study site size, ownership, treatment type present, mean annual precipitation (MAP), dominant vegetation, and range of patch sizes at each study site.

Our study sites were comprised primarily of vegetation native to their ecoregion and represented vegetation types characteristic of the southern Great Plains: gulf coastal prairie, tallgrass prairie, and shrub/mixed-grass prairie. Dominant herbaceous vegetation at the Aransas NWR (gulf coastal prairie) included *Schizachyrium scoparium*, *Sorghastrum nutans*, and *Spartina spartinae*, and dominant woody plants were *Prosopis glandulosa* and *Quercus virginiana*. Sampling was conducted under Special Use Permit #21530-15-06-DI and #21530-16-25. Herbaceous vegetation at the Attwater’s Prairie-Chicken NWR (gulf coastal prairie) primarily consisted of *Schizachyrium scoparium*, *Sorghastrum nutans*, and *Panicum virgatum*, with limited woody plant presence [37]. Sampling at the Attwater’s Prairie-Chicken NWR was performed under Special Use Permit #ATW-14-004. The Tallgrass Prairie Preserve herbaceous community was dominated by *Andropogon gerardii*, *Schizachyrium scoparium*, and *Sorghastrum nutans*. Woody plants at the site included *Quercus marilandica* and *Q*. *stellata* [26, 38, 39]. Vegetation sampling at Tallgrass Prairie Preserve was performed under a verbal agreement. Herbaceous plants at the Packsaddle WMA included *Schizachyrium scoparium*, *Andropogon gerardii*, and *Bouteloua curtipendula*, among others [40]. *Quercus havardii* was the dominant woody plant, but some areas were comprised primarily of *Artemisia filifolia* [40]. Vegetation sampling at Packsaddle WMA was conducted under a verbal agreement. Sites were located across a precipitation gradient from semi-arid at the westernmost site to wet and highly productive in the east. Climates vary across sites from temperate at the northernmost site to humid subtropical at the southernmost. Other differences between study sites included ownership (e.g., federal, state), soils, length of time under current management, and scale of treatment. Prairie-chickens were not handled or disturbed during the course of vegetation sampling.

### Vegetation sampling

We collected vegetation samples from June 2014 through August 2016. At each study site, patches treated with pyric-herbivory and fire-only were identified. To avoid effects of potentially confounding factors, patches were limited to those without a known history of other (e.g., mechanical or chemical) landscape-scale treatments. If multiple patches at a study site were burned in the same year and season (growing vs dormant), the patch to be sampled was randomly selected using a random number generator. Eight permanent 25 m transects were randomly placed in each patch. In an effort to avoid vegetation differences caused by variability in fire intensity between headfires, backfires, and flank-fires [41], transects were greater than 50 meters from patch perimeters, roads, or natural fire breaks. Transects were randomly placed in both pyric-herbivory and fire-only patches with an attempt to focus on patches with similar-time-since fire (months) at each study site. If a patch was burned during the course of the study, transects within the patch continued to be sampled, with months since fire reset corresponding to the most recent fire. This allowed us to create a chronosequence of the vegetation response. Because of the high fire frequency at all sites, few patches reached 48 months post-fire. Therefore, we focused sampling on patches with less than 48 months post-fire.

We measured vegetation characteristics at 5-m intervals (excluding 0) in 0.25 m^2^ plots along each transect. Vegetation was sampled throughout the year and relative to occurrence of fire for both summer and winter fires. Sampling times post-fire were targeted for less than 6 months, 6–12 months, 12–24 months, 24–36 months and 36–48 months. Vegetation variables measured included aboveground biomass (kg per ha), mean height (cm) of woody and herbaceous vegetation, height (cm) of tallest woody and herbaceous vegetation, and percent cover of grasses, forbs, shrubs, bare ground, and litter. To avoid artificially altering future vegetation measurements along transects, we clipped biomass (live and residual) from five 0.25 m^2^ plots ten m away from each transect. Percent cover of functional groups (grass, shrub, forb) was recorded within each plot using the Daubenmire cover classification method [42]. Vegetation height was measured using a wooden dowel rod 12 mm in diameter and 122 cm in length, painted white and marked at 1 cm increments. Using this rod, we also recorded the highest point at which live and dead/dormant vegetation touched the pole at 4 random locations within each sample plot [43].

### Analysis

We first tested for correlations between all vegetation variables to check for multicollinearity (r > |0.8|). Because of high correlation between the height of tallest shrub and mean shrub height (r = 0.96, p<0.001), height of tallest shrub was dropped from further analyses. Due to the proportional nature of vegetation cover variables, we used generalized linear mixed models (glmer) in package lme4 of the R environment to compare fire-only to pyric herbivory treatments [44, 45]. These variables were recorded as proportional data between 0 and 1, and we used a poisson distribution with a logit link function to fit models [46]. To compare biomass and height variables, we used linear mixed effects (lmer) models using the same R package [44]. For each vegetation variable, *a priori* models were created to assess the effects of time in months since fire (MSF), grazing, and the fire-grazing interaction. To account for spatial autocorrelation within sites, we included site as a nested random variable [46]. The inclusion of site as a random variable also allowed us to evaluate the vegetation patterns across a large spatial scale and compare treatments rather than sites. Collection year was included as a crossed random factor to account for potential differences in large-scale weather patterns across years.

## Results

Over the course of our study we collected vegetation measurements and biomass samples from 3,190 points across 40 unique patches and 311 transects (S1 Table). Of these sampling points, 1,510 (47.3%) were located in pyric herbivory treatments and 1,680 were in fire-only treatments. Due to the nature of the “shifting mosaic” created by non-static burn units and the fact that a few units surpassed our time-since-fire threshold of 48 months, not all patches were sampled an equal number of times.

### Vegetation characteristics

**Fig 2:**
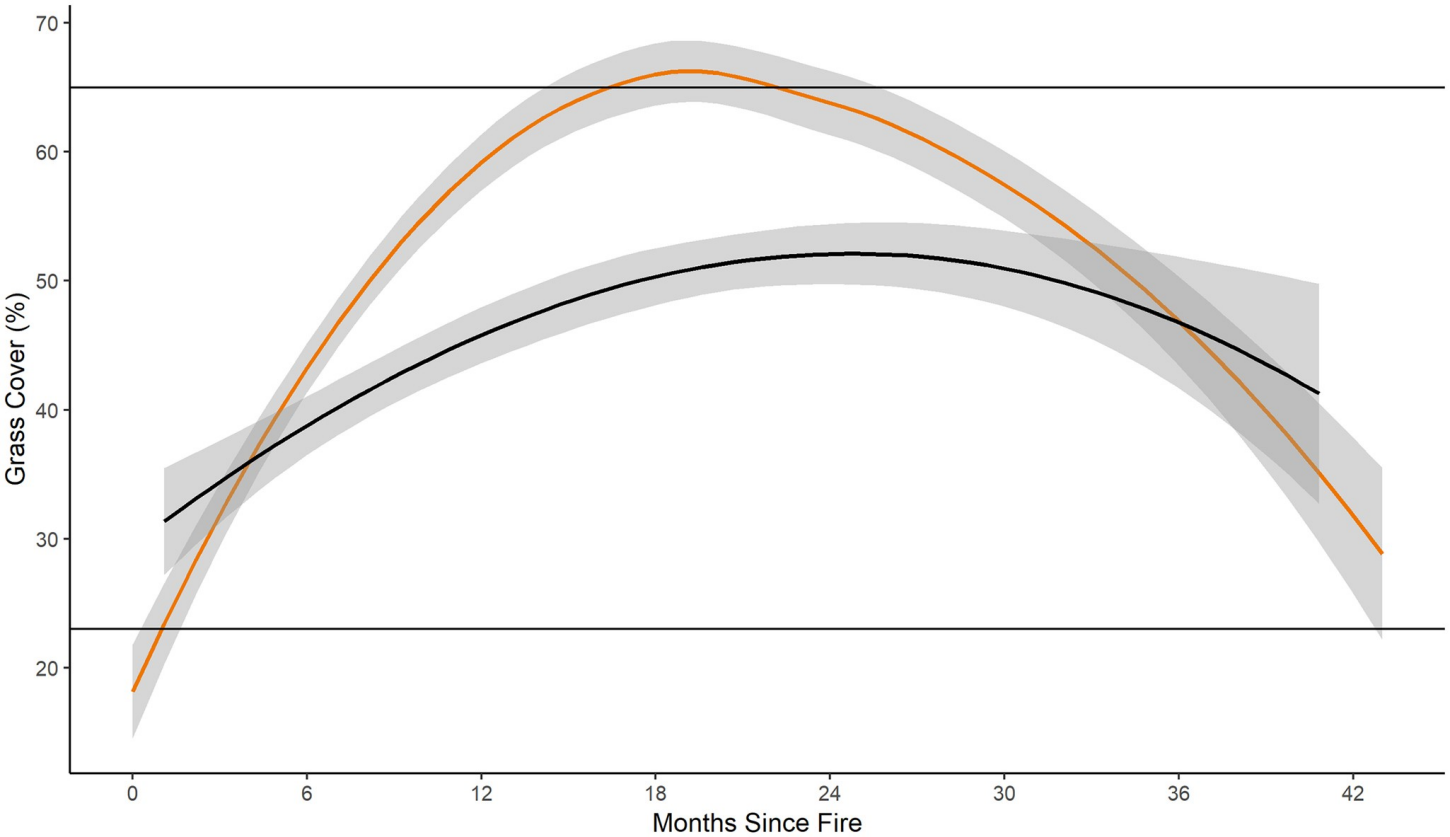
Percent grass cover. Percent grass cover for fire-only (orange) and pyric herbivory (black) treatments at all sites from 2014 to 2016. Shaded area indicates 95% confidence interval. Horizontal lines represent the minimum (~23%) and maximum (~65%) grass cover reported used for nesting by *Tympanuchus* spp. *Regression lines and confidence intervals generated using “loess” locally-weighted smoothing function.

**Fig 3:**
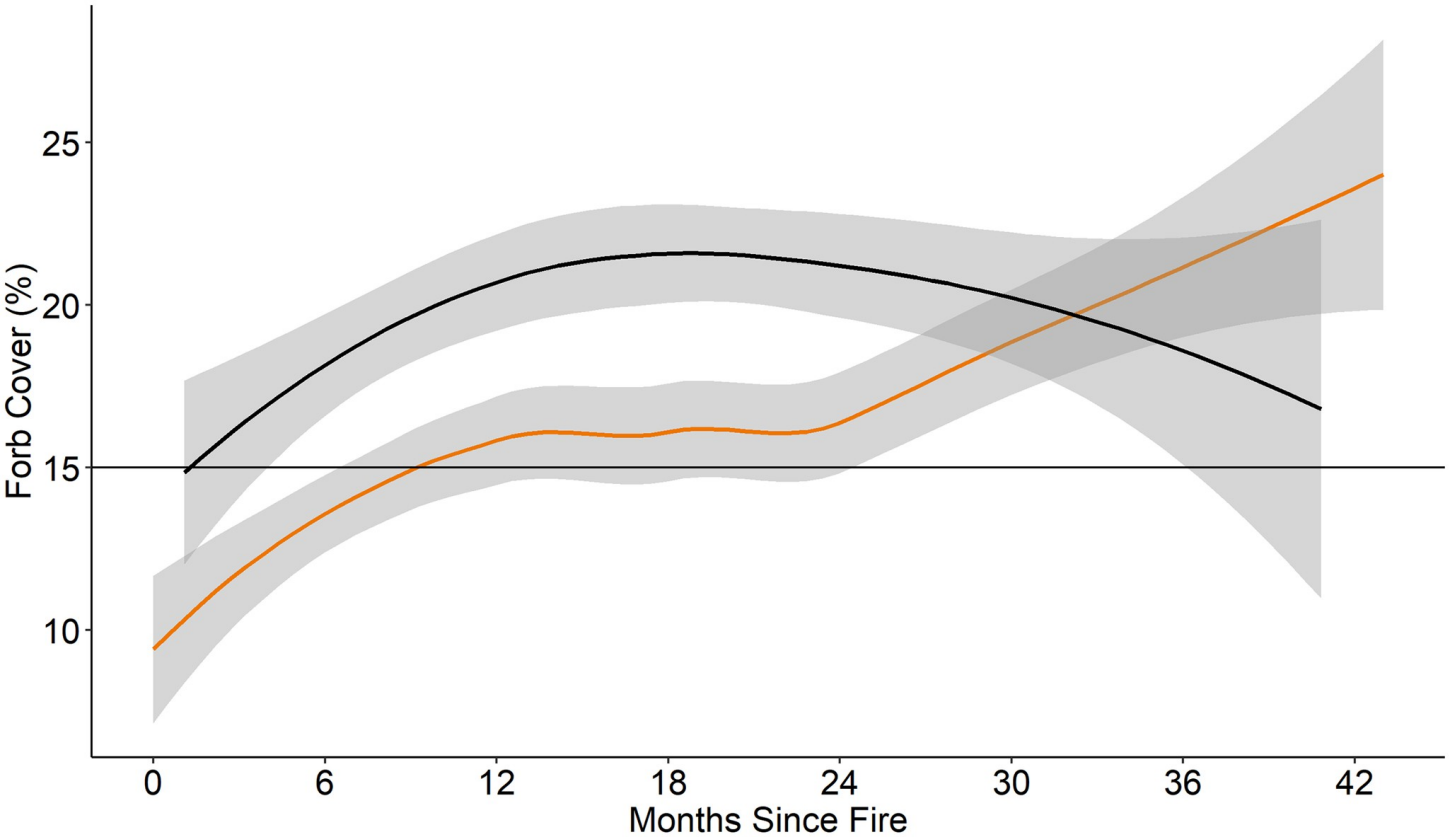
Percent forb cover. Percent forb cover for fire-only (orange) and pyric herbivory (black) treatments at all sites from 2014 to 2016. Shaded area indicates 95% confidence interval. Horizontal line represents minimum cover of forbs (15%) selected by females with broods as described in primary literature. *Regression lines and confidence intervals generated using “loess” locally-weighted smoothing function.

**Fig 4:**
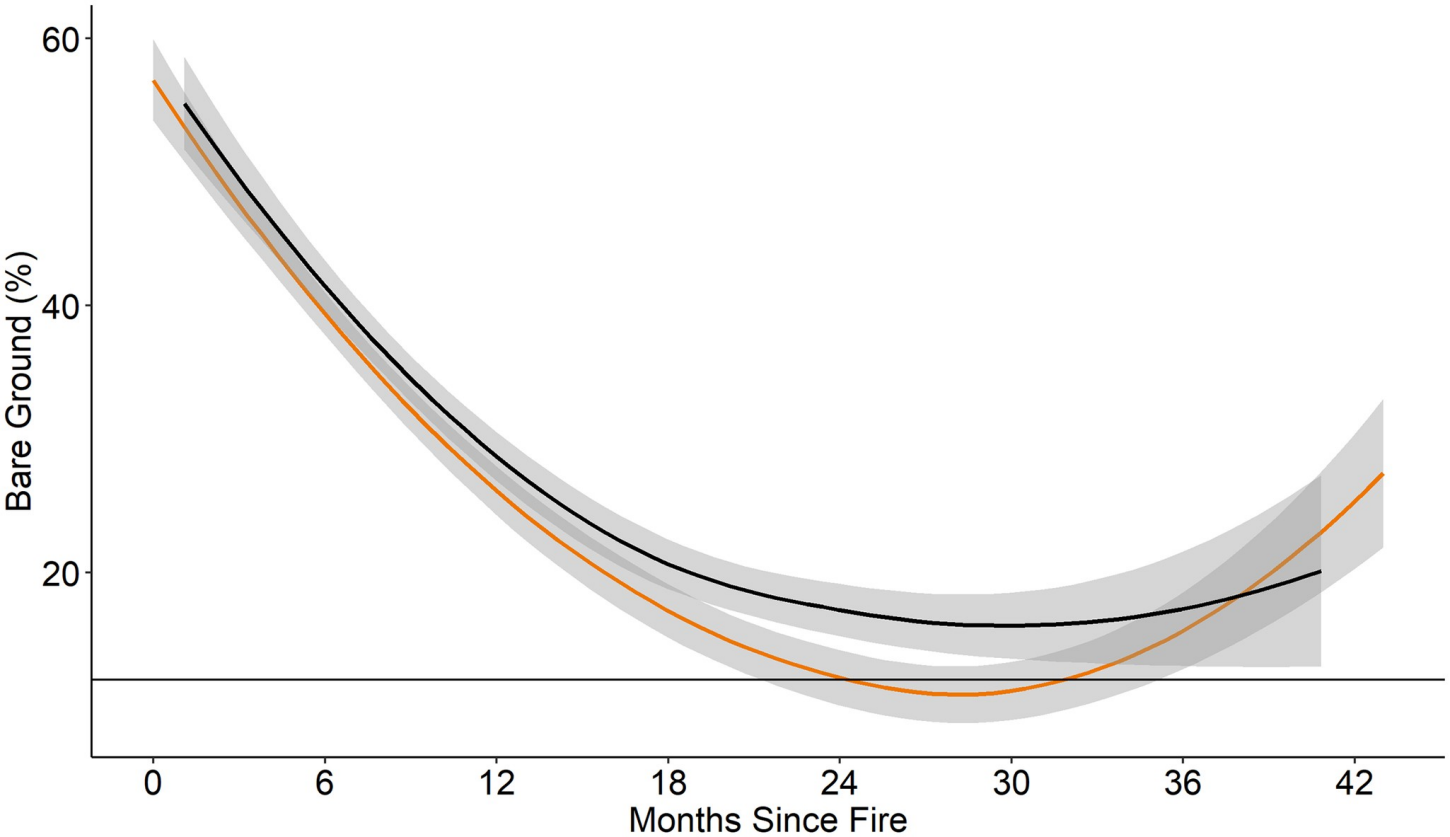
Percent bare ground. Percent bare ground for fire-only (orange) and pyric herbivory (black) treatments at all sites from 2014 to 2016. Shaded area indicates 95% confidence interval. Horizontal line represents the minimum amount of bare ground (12%) reported for areas used by prairie-chicken broods. *Regression lines and confidence intervals generated using “loess” locally-weighted smoothing function.

Time-since-fire was a significant predictor of all vegetation cover (composition) variables we recorded (Table 3). The fire-grazing interaction of pyric-herbivory was a significant predictor of percent grass cover, percent forb cover, and percent litter cover (Table 3). Grass cover (%) was greater in fire-only treatments than in the pyric herbivory treatment beginning approximately 6 months post-fire (Fig 2). Grass cover (%) increased at a greater rate in fire-only than in pyric herbivory treatments (Fig 2). Forbs had greater percent cover in pyric herbivory than fire-only treatments for more than 24 months post-fire (Fig 3). Percent bare ground was higher in pyric herbivory than fire-only treatments between 18 and 30 months post-fire (Fig 4). Bare ground reached a minimum of approximately 7% in fire-only treatments, lower than the minimum recommendation of 12% for brooding greater prairie-chickens and 36% bare ground used by lesser prairie-chicken broods [47, 48].

**Table 3:** Treatment effects on vegetation.

	Fixed effects								
	Interaction effects (MSF*Grazing)			Main effects (MSF)			Main effects (Grazing)		
Response (Cover)	*β*	*σ*	p	*β*	*σ*	p	*β*	*σ*	p
Grass cover (%)	**0.033**	**0.016**	**<0.05**	**0.113**	**0.011**	**<0.001**	-0.970	0.653	0.14
Forb Cover (%)	**-0.035**	**0.012**	**<0.01**	**0.031**	**0.008**	**<0.001**	**0.911**	**0.239**	**<0.001**
Shrub cover (%)	0.013	0.023	0.57	**0.042**	**0.015**	**<0.01**	0.013	0.023	0.16
Bare ground (%)	0.003	0.018	0.85	**-0.149**	**0.018**	**<0.001**	0.437	0.519	0.4
Litter (%)	**0.060**	**0.020**	**<0.001**	**0.029**	**0.013**	**<0.05**	**-1.267**	**0.472**	**<0.01**
Response (Structure)									
Tall Herbaceous (cm)	**1.067**	**0.157**	**<0.001**	**1.690**	**0.131**	**<0.001**	**-35.644**	**9.333**	**<0.001**
Mean Herbaceous (cm)	**0.523**	**0.097**	**<0.001**	**1.054**	**0.082**	**<0.001**	**-19.104**	**5.650**	**<0.01**
Mean Shrub (cm)	**0.184**	**0.092**	**<0.05**	-0.126	0.068	0.060	-6.115	2.994	0.051
Max Live (cm)	**0.223**	**0.088**	**<0.05**	**1.586**	**0.076**	**<0.05**	**-16.426**	**6.970**	**<0.05**
Max Dead (cm)	-0.036	0.077	0.637	**1.027**	**0.062**	**<0.001**	**-7.712**	**2.947**	**<0.05**

Regression coefficients, standard errors, and significance levels for fixed effects of the fire-grazing interaction and the main effects of months since fire (MSF) and grazing. Bold text indicates significance at the α < 0.05 level.

**Fig 5:**
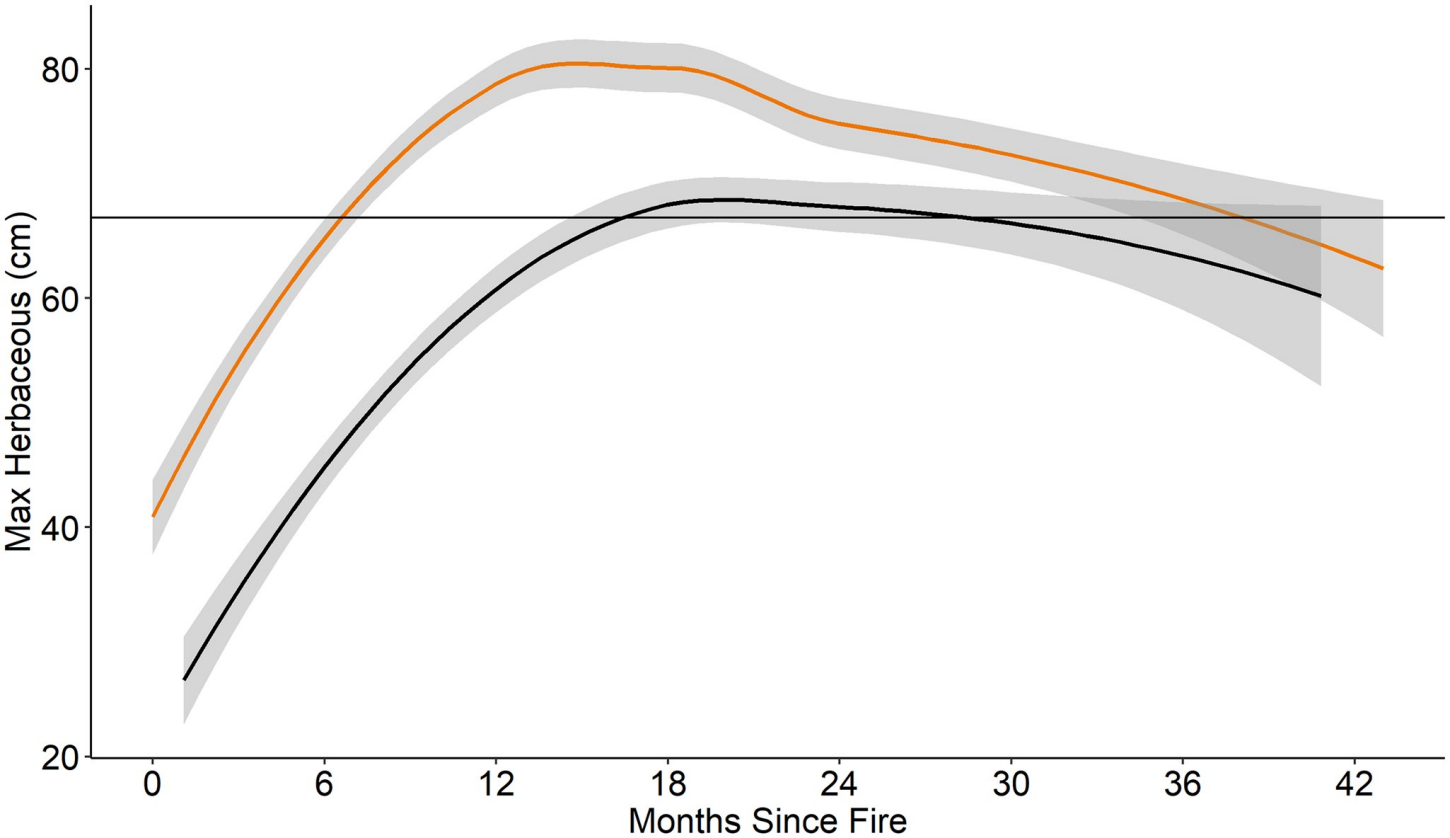
Maximum herbaceous vegetation. Maximum height (cm) of herbaceous vegetation in fire-only (orange) and pyric herbivory (black) treatments at all sites from 2014 to 2016. Shaded area indicates 95% confidence interval. Horizontal line represents maximum mean herbaceous vegetation height (67 cm) reported for prairie-chicken nest selection, as found in primary literature. *Regression lines and confidence intervals generated using “loess” locally-weighted smoothing function.

**Fig 6:**
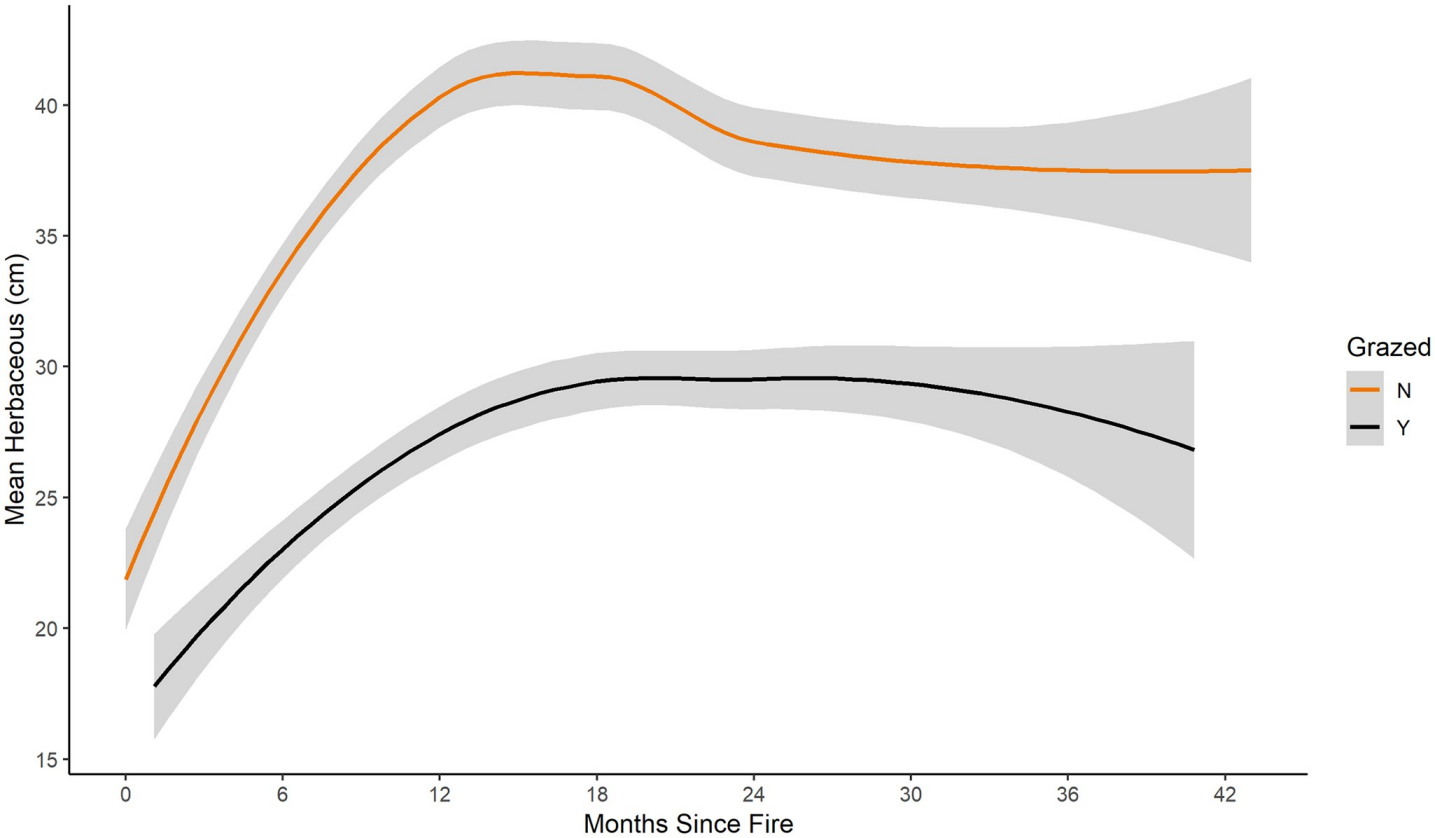
Mean herbaceous vegetation. Mean height (cm) of herbaceous vegetation in fire-only (orange) and pyric herbivory (black) treatments at all sites from 2014 to 2016. Shaded area indicates 95% confidence interval. *Regression lines and confidence intervals generated using “loess” locally-weighted smoothing function.

**Fig 7:**
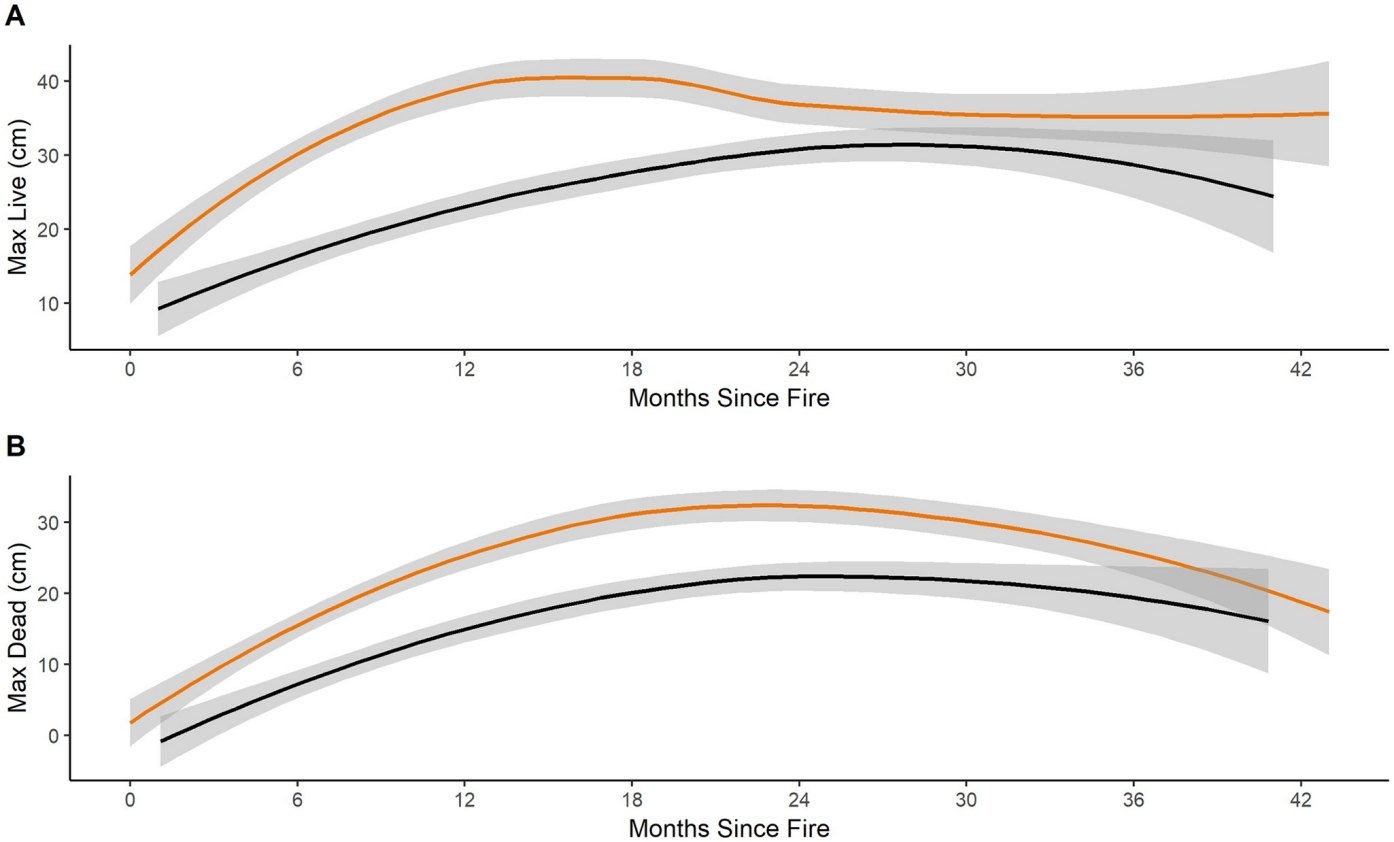
Maximum live and dead vegetaton. Maximum heights (cm) of live (A) and dead (B) vegetation in fire-only (orange) and pyric herbivory (black) treatments at all sites from 2014 to 2016. Shaded area indicates 95% confidence interval. *Regression lines and confidence intervals generated using “loess” locally-weighted smoothing function.

**Fig 8:**
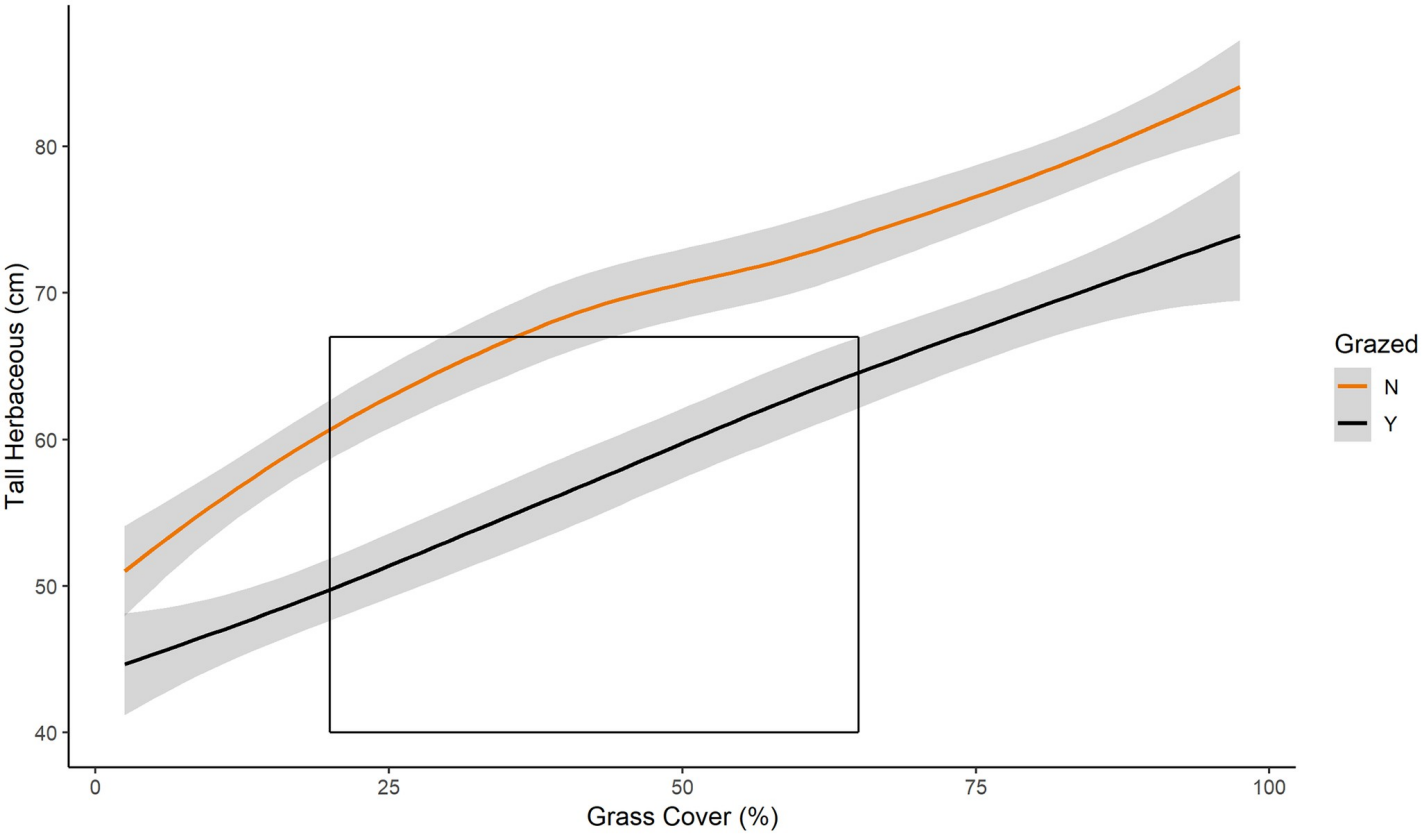
Vegetation height vs percent grass. Maximum height(cm) of herbaceous vegetation related to percent cover of grasses in fire-only (orange) and pyric herbivory (black) treatments at all sites from 2014 to 2016. Area within box represents the minimum and maximum recommended parameters for suitable nesting cover for prairie-chickens from primary literature. Shaded area indicates 95% confidence interval. *Regression lines and confidence intervals generated using “loess” locally-weighted smoothing function.

Time-since-fire was a statistically significant positive predictor of all vegetation structure variables we measured, with the exception of mean shrub height (p = 0.07). The fire-grazing interaction was a significant predictor with all vegetation structural variables, except maximum height of dead/dormant vegetation (p = 0.637). Maximum and mean height of herbaceous vegetation were lower in pyric herbivory than in fire-only treatments (Figs 5 and 6, respectively). Additionally, mean height of both live and dead vegetation were greater in fire-only than in pyric herbivory treatments (Fig 6). Our results also revealed treatment differences in the relationship between maximum height of herbaceous vegetation and percent grass cover. Maximum herbaceous vegetation was taller in fire-only treatments as percent cover of grasses increased (Fig 8), a relationship consistent with the “grazing lawns” produced by pyric herbivory. Maximum height of herbaceous vegetation in pyric herbivory treatments rose above the maximum threshold reported for use by prairie-chickens nearly 18 months post-fire, whereas fire-only treatments crossed the same threshold at approximately 6 months post-fire (Fig 5). Likewise, maximum heights of live and dead/dormant vegetation remained lower than in pyric herbivory treatments than fire-only treatments (Fig 7). The maximum height of herbaceous vegetation across sites in our study in fire-only areas was approximately 80 cm, compared to approximately 67 cm in pyric herbivory treatments (Fig 5).

### Site variability

The random effects coefficients from our models revealed that most of the variation due to site characteristics occurred within, rather than across sites (Table 4). For maximum height of herbaceous vegetation, 49.7% of the random variation was a function of differences at the patch or transect (within-site) level. For mean herbaceous vegetation height, 48.2% of the random variation occurred at the within-site level. Similarly, patch-level variation accounted for 62.7% of the random variation in maximum live vegetation height. For mean shrub height, the only variable not affected by time-since-fire, patch-level variation accounted for just 12.2%, while residual (unexplained) variation was responsible for 64% of the random variation. Residual variation was also considerable for maximum dead/dormant vegetation height, the only vegetation structure variable not affected by the fire-grazing interaction. Variation due to year ranged from 0.3% for mean shrub height to 12% for maximum height of dead/dormant vegetation.

**Table 4:** Variance components.

	Variance Components (Random Effects)									
	Transect		Patch		Site		Year		Residual	
	*σ*^2^	%	*σ*^2^	%	*σ*^2^	%	*σ*^2^	%	*σ*^2^	%
Tall Herbaceous (cm)	76.050	5.195	728.000	49.734	21.800	1.489	55.830	3.814	582.100	39.767
Mean Herbaceous (cm)	21.000	3.584	282.620	48.229	0.000	0.000	59.880	10.218	222.500	37.969
Mean Shrub (cm)	27.003	7.363	44.706	12.191	58.504	15.954	1.089	0.297	235.413	64.195
Max Live (cm)	18.970	2.646	449.290	62.662	0.000	0.000	67.960	9.478	180.790	25.214
Max Dead (cm)	33.980	12.075	63.590	22.598	0.000	0.000	33.520	11.912	150.310	53.415

Random effects variance σ^2^ and percent of total variance for random effects at multiple levels within and across sites as well as year.

## Discussion

Our data suggest pyric herbivory has significant effects on vegetation community composition and structure. Likewise, the significant effects we observed as a result of the fire-grazing interaction as compared to fire only highlight the importance of complex interactions between biotic (e.g., herbivory) and abiotic (e.g. fire) factors in shaping the landscape in which prairie-chickens evolved. Our results indicate that time-since-fire and its interaction with grazing are major drivers of vegetation functional group composition and structure and have direct implications to prairie-chicken habitat.

Prairie-chickens have been shown to select for areas with a high percentage (>40%) of bare ground for use as lekking sites [19]. Our data reveal that both fire-only and pyric herbivory treatments offer greater than 40% bare ground for up to approximately 6 months post-fire. In addition to bare ground, leks are generally found in areas with vegetation less than 16 cm in height [19]. The fire-grazing interaction forms “grazing lawns,” areas of very short (10–15 cm) vegetation cover resulting from intense grazing pressure by herbivores [34, 49]. These grazing lawns are maintained until the next patch is burned, which then itself becomes a grazing lawn. In a landscape managed with pyric herbivory, the presence of grazing lawns offers lekking areas that shift in space and time as a result of the fire-grazing interaction [38]. In the absence of this shifting mosaic, leks are often fairly static in space and may be associated with shallow soil areas or cattle point attractants such as water and minerals [50, 51].

In addition to bare ground, prairie-chicken females with broods select for areas with moderate canopy cover (~25–60%, 20–30 cm tall) as well as sufficient bare ground [17, 19]. Considering these recommendations, our results indicate that pyric herbivory treatments provide potential brooding habitat for at least 48 months, whereas fire-only units exceeded 60% canopy cover within 12 months. Broods of greater prairie-chickens use areas with a high proportion of bare ground, but brood survival tends to be a function of forb cover [52]. Our results suggest that pyric herbivory increases or extends the length of time both are present compared to fire-only treatments, thereby extending the amount of time areas provide available habitat. Between 18 and 30 months post-fire, bare ground was greater in pyric herbivory treatments, with forb cover higher until approximately 30 months. The difference in bare ground indicates that pyric herbivory may improve the use of patches with greater time-since-fire for prairie-chicken broods compared to fire-only treatments. Therefore, our data suggest pyric herbivory can provide prairie-chicken brood habitat for up to 30 months post-fire.

Our results indicate that the vegetation structure created by pyric herbivory maintains vegetation heights below that which is avoided by nesting prairie-chickens. Moreover, the vegetation structural characteristics we observed are within the range of values recommended for providing nesting cover [17, 19], and nesting cover in pyric herbivory treatments remained within this range for a longer time than in fire-only treatments. Nesting cover for prairie-chickens consists of grasslands comprised of vegetation 15–67 cm in height, usually dominated by senescent material from prior years’ growth [19]. Lesser prairie-chicken nests in sand-sagebrush mixed-grass ecosystem have been reported in cover 48 cm tall, with total (grass, forb, and shrub) canopy cover greater than 60% [53]. In a grassland system, lesser prairie-chickens selected sites with ~65% grass cover, ~20% forb cover, and ~10% bare ground [25]. Nesting Attwater’s prairie-chickens have been reported to select areas of vegetation that averaged 67 cm in height, but avoid taller vegetation [27].

Fire-only treatments did provide habitat characteristics within suggested parameters to meet requirements of prairie-chickens. In some instances, fire-only treatments may provide “better” habitat characteristics than pyric herbivory for some period of time post-fire. For example, the relationship between maximum height and percent cover of herbaceous vegetation has been reported to play a role in nest site selection and nest success [17]. Because the herbivory component of pyric herbivory treatments reduces the rate at which vegetation height and percent herbaceous cover increase, fire-only treatments may provide nesting habitat characteristics sooner than pyric herbivory treatments. In an ideal landscape, a mosaic of pyric herbivory and fire-only treatments would shift spatially and temporally to provide critical habitat characteristics for prairie-chickens as well as other fauna.

Despite the differences among our study sites, our results suggest that the importance of time-since-fire and the fire-grazing interaction can reasonably be generalized across the southern Great Plains. Considerable variation in random effects at the site level would have indicated that unmeasured site differences contributed significantly to our results. However, that the largest source of variation due to site factors came from within-site differences illustrates the applicability of our results to rangelands of the southern Great Plains. Thus, restoration of pyric herbivory, implemented at a landscape scale, appears to offer significant conservation opportunities for prairie-chicken management. Previous research has concluded the same for the conservation of other grassland birds [9]. The effect of pyric herbivory should be expected to differ depending upon cattle stocking rate and size of management patches relative to the landscape being managed [16, 31]. For instance, our overall results did not reveal sustained presence of grazing lawns, a finding which may be the result of site differences in patterns of fire implementation. At the APCNWR, burns are executed on numerous relatively small plots (20–400 ha), often within a single week. Such a pattern, coupled with low-moderate livestock stocking rates, may effectively reduce the intensity of grazing to a degree that grazing lawns cannot be achieved. Reduced grazing intensity allows for more rapid increases in biomass, reducing the effectiveness of fuels reduction treatments as well.

## Management implications

Landscapes managed for prairie-chicken conservation in the highly productive southern Great Plains should strive to maximize heterogeneity to provide vegetation structure for lekking, nesting, and brood-rearing. Time-since-fire and pyric herbivory are significant drivers of vegetation structure and composition. However, vegetation response to pyric herbivory differs from fire-only as illustrated by our data. Using pyric herbivory as a systemic land management strategy across multiple ecoregions can benefit prairie-chicken conservation by providing vegetation for lekking, nesting, and brooding cover within close proximity both spatially and temporally. Considering that much of the rangeland in the southern Great Plains is privately owned and used for livestock production, it is important to note that pyric herbivory is compatible with livestock production. This compatibility ensures that producers can manage toward achieving conservation and production goals simultaneously. In addition to benefitting conservation of the declining *Tympanuchus* genus, restoring heterogeneity to Great Plains landscapes using pyric herbivory has been repeatedly illustrated to offer benefits to biodiversity.

## Supporting information

S1 Table (XLSX)
